# Neoadjuvant Treatment in Patients With Resectable and Borderline Resectable Pancreatic Cancer

**DOI:** 10.3389/fonc.2020.00041

**Published:** 2020-01-31

**Authors:** Quisette P. Janssen, Eileen M. O'Reilly, Casper H. J. van Eijck, Bas Groot Koerkamp

**Affiliations:** ^1^Department of Surgery, Erasmus MC University Medical Center, Rotterdam, Netherlands; ^2^Department of Medicine, Memorial Sloan Kettering Cancer Center, New York, NY, United States; ^3^David M. Rubenstein Center for Pancreatic Cancer Research, New York, NY, United States

**Keywords:** pancreatic cancer, neoadjuvant, FOLFIRINOX, borderline resectable, pancreatic ductal adenocarcinoma, resectable, ongoing trials, evidence

## Abstract

Approximately 20% of pancreatic ductal adenocarcinoma (PDAC) patients have (borderline) resectable pancreatic cancer [(B)RPC] at diagnosis. Upfront resection with adjuvant chemotherapy has long been the standard of care for these patients. However, although surgical quality has improved, still about 50% of patients never receive adjuvant treatment. Therefore, recent developments have focused on a neoadjuvant approach. Directly comparing results from neoadjuvant and adjuvant regimens is challenging due to differences in patient populations that influence outcomes. Neoadjuvant trials include all patients who have (B)RPC on imaging, while adjuvant-only trials include patients who underwent a complete resection and recovered to a good performance status without any evidence of residual disease. Guidelines recommend neoadjuvant treatment for BRPC patients mainly to improve negative resection margin (R0) rates. For *resectable* PDAC, upfront resection is still considered the standard of care. However, theoretical advantages of neoadjuvant treatment, including the increased R0 resection rate, early delivery of systemic therapy to all patients, directly addressing occult metastatic disease, and improved patient selection for resection, may also apply to these patients. A systematic review by intention-to-treat showed a superior median overall survival (OS) for any neoadjuvant approach (19 months) compared to upfront surgery (15 months) in (B)RPC patients. A neoadjuvant approach was recently supported by three randomized controlled trials (RCTs). For resectable PDAC, neoadjuvant treatment was superior in a Japanese RCT of neoadjuvant gemcitabine with S-1 vs. upfront surgery, with adjuvant S-1 in both arms (median OS: 37 vs. 27 months, *p* = 0.015). A Korean trial of neoadjuvant gemcitabine-based chemoradiotherapy vs. upfront resection in BRPC patients was terminated early due to superiority of the neoadjuvant group (median OS: 21 vs. 12 months, *p* = 0.028; R0 resection: 52 vs. 26%, *p* = 0.004). The PREOPANC-1 trial for (B)RPC patients also showed favorable outcome for neoadjuvant gemcitabine-based chemoradiotherapy vs. upfront surgery (median OS: 17 vs. 14 months, *p* = 0.07; R0 resection: 63 vs. 31%, *p* < 0.001). FOLFIRINOX is likely a better neoadjuvant regimen, because of superiority compared to gemcitabine in both the metastatic and adjuvant setting. Currently, five RCTs evaluating neoadjuvant modified or fulldose FOLFIRINOX are accruing patients.

## Introduction

Pancreatic ductal adenocarcinoma (PDAC) accounts for 3% of all new cancer diagnoses, and incidence rates continue to slowly increase. In contrast to the decreasing cancer-related death rates for many other solid organ malignancies, PDAC survival has not shown much improvement over the last decades (1). As a consequence, PDAC is expected to be the second leading cause of cancer-related death in the United States by 2030 (2). An important explanation for the high mortality rate compared to other solid tumors, is that the majority of patients are diagnosed with metastatic disease (40%) or locally advanced disease (40%). For metastatic PDAC, palliative treatment using multi-agent chemotherapy such as a combination of 5-FU, oxaliplatin, and irinotecan (FOLFIRINOX) or gemcitabine with *nab*-paclitaxel is the standard of care based on randomized controlled trials (RCTs) (3, 4). These therapies have been shown to increase life expectancy with 2–4 months. For locally advanced pancreatic cancer (LAPC), no RCT has been completed, but based on a patient-level meta-analysis and the survival benefit in metastatic PDAC, FOLFIRINOX, and gemcitabine with *nab*-paclitaxel are the standard initial treatments (5). Following induction chemotherapy, some patients will also receive chemoradiation and about 20% of LAPC patients undergoes surgical resection. The remaining 20% of PDAC patients have (borderline) resectable pancreatic cancer [(B)RPC] at diagnosis.

Resection remains the only curative-intent treatment. However, even curative-intent surgery typically does not overcome the aggressive biology, resulting in recurrent disease within 2 years after resection in the vast majority of patients (6). Studies focusing on recurrence patterns have demonstrated that the initial recurrence in 76% of patients was systemic (7, 8). Therefore, also (B)RPC could be approached as a systemic disease, irrespective of apparent non-metastatic disease on imaging (9).

The objective of this paper is two-fold. First, we aim to give a general overview of the current treatment strategies for (B)RPC patients, to discuss the rationale for neoadjuvant and adjuvant therapy, and to consider the challenges when comparing these treatment approaches. Second, we aim to summarize the currently available evidence for neoadjuvant treatment with a special focus on neoadjuvant FOLFIRINOX, including published and ongoing phase II-III trials for neoadjuvant treatment.

## Methods

To identify relevant studies for neoadjuvant treatment, a comprehensive search of Clinicaltrials, Embase, and MEDLINE was performed. Search terms included “neoadjuvant,” “FOLFIRINOX,” “folinic acid,” “fluorouracil,” “irinotecan,” “oxaliplatin,” “pancreas cancer,” “drug combination,” and relevant variants thereof. Only articles written in English were assessed. Articles were selected based on relevance for our objectives, considering methodological quality, study type, number of included patients, and additional value to current knowledge. A selection was made for prospective studies with restriction to phase II and III trials and publication dates from 2006 to 2019. Furthermore, references of included articles were assessed for additional relevant literature.

### Disease Staging

Non-metastatic pancreatic cancer is subdivided into *resectable* PDAC, BRPC, and LAPC. Historically however, BRPC was not recognized as a unique disease stage. In 2001, a first definition of marginally resectable tumors was proposed (10). The term “borderline resectable” was thereafter introduced by the 2006 National Comprehensive Cancer Network (NCCN) guidelines for tumors at risk for margin-positive resection when treated with upfront surgery, and adopted by other guidelines. The critical aspects that need to be evaluated are the contact of the tumor with the superior mesenteric vein or portal vein complex (SMV-PVC) as venous structures, and the superior mesenteric artery (SMA), common hepatic artery (CHA), and celiac artery (CA) as major surrounding arteries. Over time, several criteria have been proposed to define resectability status, summarized in Table 1.

**Table 1:** Comparison of imaging-based criteria distinguishing resectable, borderline resectable, and locally advanced pancreatic cancer.

	MD Anderson (2008) (11, 12)^*^	**AHPBA/SSAT/SSO (2009) (13)**	ISGPS (2014)^**^(14)	NCCN (2019)^***^ (15)
**RESECTABLE PANCREATIC CANCER**				
SMA	No contact			
CHA				
CA				
SMV—PVC	Patent	No abutment, distortion, thrombus, or encasement	No distortion	No contact or ≤180° without vein contour irregularity
**BORDERLINE RESECTABLE PANCREATIC CANCER**				
SMA	≤180°			
CHA	≤180° or short-segment encasement (>180°) without extension to celiac axis or hepatic artery bifurcation, allowing for safe and complete resection and reconstruction	Encasement of gastroduodenal artery up to CHA with short segment encasement or direct abutment of CHA without extension to celiac axis		Contact without extension to celiac axis or hepatic artery bifurcation, allowing for safe and complete resection and reconstruction.
CA	≤180°	No abutment or encasement		≤180° or (for corpus) >180° without aortic involvement and intact gastroduodenal artery permitting modified Appleby procedure.
SMV—PVC	Segmental occlusion with possibility of reconstruction	Abutment, encasement or short-segment occlusion with possibility of reconstruction	Distortion, narrowing, or occlusion with possibility of reconstruction	>180° or ≤180° with contour irregularity or occlusion with possibility of complete resection and reconstruction, or solid tumor contact with inferior vena cava.
**LOCALLY ADVANCED PANCREATIC CANCER**				
SMA	>180°			
CHA	≤180° or >180° with extension to celiac axis, splenic or left gastric junction	Encasement of gastroduodenal artery up to CHA with short segment encasement or direct abutment of CHA with extension to celiac axis		Contact with extension to celiac axis or hepatic artery bifurcation
CA	>180°	Abutment or encasement and technically not reconstructable	Abutment, or any contact with aortic involvement	>180° or any contact with aortic involvement
SMV—PVC	Occluded or encased and technically not reconstructable			Unreconstructable duo to tumor involvement or occlusion, or contact with most promixal draining jejunal branch into SMV

*SMA, superior mesenteric artery; CHA, common hepatic artery; CA, celiac artery; SMV—PVC, superior mesenteric vein—portal vein complex; AHPBA/SSAT/SSO, Americas Hepato-Pancreato-Biliary Association/Society of Surgical Oncology/Society for Surgery of the Alimentary Tract; NCCN, National Comprehensive Cancer Network*.

* *Patients with poor functional status and/or severe medical comorbidities (type C), as well as those with technically resectable disease but with imaging studies suspicious for metastatic disease (type B) are also classified as borderline resectable*.

** *The ISGPS criteria were adopted by the 2013 NCCN criteria*.

*** *The NCCN criteria have changed over the years. The most recent criteria (3.2019) are included*.

Commonly used criteria include the NCCN guidelines (15, 16), MD Anderson Cancer Center (MDACC) guidelines (11, 12), the AHPBA/SSAT/SSO expert consensus guidelines (13), and the International Study Group of Pancreatic Surgery (ISGPS) criteria (14). The 2013 NCCN guidelines adopted the ISGPS criteria, and minor modifications were made in the following NCCN guidelines. The AHPBA/SSAT/SSO guidelines require less vascular abutment to classify patients as BRPC compared to the NCCN and MDACC guidelines. For example, tumors with any SMV-PVC abutment are BRPC in the AHPBA/SSAT/SSO guidelines. In contrast, the other two guidelines require venous occlusion (MDACC) or vein contour irregularity (NCCN), regardless of the extent of abutment of the tumor with the SMV-PVC.

Several factors associated with these criteria have complicated comparison of study outcomes. First, no uniformly accepted set of criteria exists. Second, the NCCN guidelines have been modified several times. Third, most guidelines include ambiguous terms to define the resectability stages, including “abutment, impingement, involvement, and encasement.” The classifications are based on apparent contact on imaging of tumor and blood vessel. The actual presence of tumor cells surrounding the vessels (or invading the vessel wall) is rarely known before pathological examination of the resected specimen. However, patients with extensive apparent contact on imaging often undergo a surgically incomplete (R1) resection, suggesting imaging is indeed a good predictor of the presence of tumor cells surrounding and/or invading the vessel wall. Lack of international agreement on the definition of an R0 resection (i.e., >1 vs. >0 mm) and standardized protocols for pathological examination (i.e., axial slicing vs. bivalving) may explain variation in published R0 resection rates (17, 18). At a consensus meeting in 2016, it has been proposed to add biological and functional risk factors to the resectability criteria. Biological factors include elevated Carbohydrate Antigen (CA) 19.9 levels above 500 units/mL, regional lymph node metastases, and suspicion of distant metastases without the possibility for pathological proof. The functional factors include performance status and comorbidity (19). These biological and functional factors have also been implemented in the NCCN 2018 and American Society of Clinical Oncology (ASCO) 2019 guidelines, further decreasing the number of patients classified as *resectable* PDAC (20, 21). Similarly, within the MDACC guidelines, three sub-types of BRPC are distinguished; based on local tumor-artery contact (type A), based on tumor marker levels or imaging suggestive of metastatic disease but lacking pathological proof (type B), or based on marginal performance status prior to treatment (type C) (11, 12).

### Adjuvant Treatment—Practice Changing Trials

Upfront surgery followed by adjuvant chemotherapy has long been the standard of care for patients with potentially *resectable* PDAC. Initial adjuvant treatment strategies included both chemotherapy and radiotherapy. In 2004, the long-term results from the ESPAC-1 (European Study Group for Pancreatic Cancer) trial were published (22). This multicenter European collaboration used a 2 × 2 factorial design to compare adjuvant 5-FU-based chemoradiotherapy alone (arm A, *n* = 73), adjuvant 5-FU based chemoradiotherapy followed by 5-FU (arm B, *n* = 72), adjuvant 5-FU alone (arm C, *n* = 75), and observation alone (arm D, *n* = 69). The trial was not powered for a direct comparison of the four groups, yet survival was longer in patients who received chemotherapy compared to patients who did not [median OS: 20 vs. 16 months, hazard ratio (HR) 0.71, *p* = 0.009]. Furthermore, comparison of patients with or without chemoradiotherapy showed *inferior* median OS for patients who received chemoradiotherapy (median OS: 16 vs. 18 months, HR 1.28, 95% CI: 0.99–1.66, *p* = 0.05). The CONKO-001 (Charité Onkologie 001) trial found that adjuvant gemcitabine was superior to observation alone with a 5-year survival rate of 21 vs. 10% (*p* = 0.01) (6). In 2017, the ESPAC-4 trial included 730 patients comparing gemcitabine (*n* = 366) to gemcitabine plus capecitabine (*n* = 364) (23). Median OS was 26 months with gemcitabine alone and 28 months with gemcitabine plus capecitabine (HR 0.82, 95% CI: 0.68–0.98, *p* = 0.032). In 2018, the results of the PRODIGE 24/CCTG PA.6 trial comparing adjuvant gemcitabine to modified FOLFIRINOX (mFOLFIRINOX) exceeded expectations (24). The median OS was 54 months with mFOLFIRINOX compared to 35 months with gemcitabine (stratified HR 0.64, 95% CI: 0.48–0.86, *p* = 0.003). mFOLFIRINOX is currently the best adjuvant treatment for patients with a good performance score.

### Neoadjuvant Treatment—Rationale

The strategy of chemotherapy following surgery has several drawbacks. First, approximately 20% of patients with (B)RPC on imaging will never undergo a resection because of occult metastatic or locally irresectable disease (25). More advanced disease is often diagnosed at exploratory laparotomy, which has considerable morbidity and mortality, and the majority of these patients will not receive any palliative chemotherapy. Even after successful resection, only about 55% of patients are able to receive adjuvant chemotherapy due to postoperative complications, clinical deterioration, or early progressive disease (26–29). Especially those patients not able to receive adjuvant chemotherapy have very poor prognosis. The CONKO-001 RCT reported that about 50% of patients in the observation arm (i.e., without adjuvant chemotherapy) hadrecurrent disease or died within 6 months after surgery; the median DFS was only 6.7 months after surgery without adjuvant chemotherapy (6). In an attempt to overcome some of these drawbacks, there is an ongoing paradigm shift toward a neoadjuvant approach. This is supported by promising results in other malignancies such as breast cancer, rectal cancer, and esophagogastric cancer (30–32). Theoretical advantages of a neoadjuvant approach are numerous. First, a much larger population can benefit from effective systemic treatment. Second, neoadjuvant systemic therapy directly addresses radiographically occult metastatic disease. Third, delaying surgery during neoadjuvant treatment allows for restaging prior to surgery. This provides improved patient selection by identifying those individuals who have responded to neoadjuvant treatment and may benefit from a resection, whilst preventing futile surgery in patients with rapidly progressive disease. Furthermore, several studies have shown that complication rates, including postoperative pancreatic fistula and postpancreatectomy hemorrhage, are lower following neoadjuvant treatment (33–36). Lastly, neoadjuvant treatment may reduce tumor volume, with increased likelihood of a margin negative (R0) resection (25, 37).

Conversely, the neoadjuvant approach has some potential drawbacks. First, patients might have progressive disease during neoadjuvant treatment, precluding curative-intent resection. However, it is unlikely that patients with progressive disease during chemotherapy would have been cured with upfront resection, since cure is exceedingly rare with a 10-year OS of only 4% after surgery (38). Furthermore, since patients with progression during neoadjuvant treatment do not seem to respond to chemotherapy, it is likely that these patients would not have responded to adjuvant chemotherapy either, increasing their risk of early recurrent or metastatic disease following surgery. Thus, rather than a missed opportunity of cure, it is more likely that these patients have been spared futile surgery. Another potential drawback is the risk of deterioration during neoadjuvant treatment. Chemotherapy may reduce the patients' performance status and quality of life because of toxicities. More specifically, FOLFIRINOX is known for its gastrointestinal complications, increased risk of infections, fatigue, and sensory peripheral neuropathy (24). Fortunately, it is rare that patients become unfit for surgery due to chemotherapy, and no deaths have been attributed to FOLFIRINOX in two systematic reviews (5, 39). A final potential drawback is that biliary drainage is required before chemotherapy in patients with obstructive jaundice. Biliary drainage is associated with mainly infectious complications (40), but this can be avoided with upfront surgery.

### Comparing Adjuvant With Neoadjuvant Trials

The PRODIGE 24/CCTG PA.6 trial showed a median survival of almost 5 years for patients with *resectable* PDAC treated with upfront resection and adjuvant mFOLFIRINOX: a survival estimate far superior than previously reported for other treatments (24). However, these results apply only to a highly selected subset of patients. Only patients with favorable tumor biology and good performance status after a complete curative-intent resection are eligible for adjuvant trials. Several hurdles need to be taken by patients with *resectable* PDAC on imaging. A small percentage of patients become unfit for surgery in the preoperative phase due to stent-related complications causing clinical deterioration. In the operative phase, a resection is not performed in about 20% of patients who are found to have occult metastatic or locally irresectable disease. Next, patients need to recover sufficiently within 12 weeks after surgery to receive adjuvant chemotherapy. In large cohorts, only about 50% of patients received adjuvant gemcitabine after a complete resection (26–29). For adjuvant mFOLFIRINOX, patients need to have an even better World Health Organization (WHO) performance status of 0 or 1. Lastly, for the PRODIGE 24/CCTG PA.6 trial, patients were ineligible if the CA 19.9 level was above 180 U/mL before start of chemotherapy or in the event of early postoperative disease recurrence on imaging. We estimate that on a nationwide level only about 25% of patients with (B)RPC on imaging could become eligible for adjuvant mFOLFIRINOX. This also explains the low accrual rate of the PRODIGE 24/CCTG PA.6 trial of only 1–2 patients on average per center per year.

Patients do not need to overcome most of these hurdles for inclusion in a neoadjuvant trial. Most patients presenting in the clinic with (B)RPC on imaging are eligible for neoadjuvant trials after adequate biliary drainage. Thus, direct comparison of outcomes of neoadjuvant and adjuvant trials is biased, because neoadjuvant trials can include almost all patients whilst for adjuvant trials only the 25% of patients with the best tumor biology and performance status can be included.

### Neoadjuvant Treatment—Systematic Reviews and Meta-Analyses

One of the first studies describing neoadjuvant treatment for pancreatic cancer was published in 1980 (41). Over time, different single-agent or multi-agent chemotherapy regimens were used, including 5-FU, gemcitabine, mitomycin C, and platinum compounds. Three large meta-analyses have been published for non-metastatic PDAC patients describing outcomes after preoperative treatment (irrespective of the regimen used) compared to upfront surgery (Table 2) (25, 37, 42). The first meta-analysis by Gillen et al. included 111 studies published from 1980 to 2009. Chemotherapy regimens were mainly gemcitabine or 5-FU based, and 94% of studies used chemoradiotherapy (42). This meta-analysis showed that 33% of patients initially staged as unresectable pancreatic cancer (BRPC and LAPC) were able to undergo a resection after preoperative treatment. Furthermore, estimated survival following resection and R0 resection rates for patients with initially unresectable PDAC were comparable to patients with *resectable* PDAC (Median OS: 23 vs. 21 months; R0 resection: 82 vs. 79%). A second meta-analysis by Dhir et al. provided an update of the literature published since 2009, which marks the endorsement of the AHPBA/SSAT/SSO consensus criteria, as well as the introduction of newer preoperative regimens (37). In this meta-analysis of 96 studies, the median OS after neoadjuvant treatment for *resectable* PDAC and BRPC was similar (18 vs. 19 months). Furthermore, the R0 resection rate of 85% was much higher than previously reported in the setting of upfront resection. The third meta-analysis by Versteijne et al. included only studies that did not exclude patients who didn't undergo resection after neoadjuvant treatment or patients who didn't undergo adjuvant chemotherapy after resection (25). These criteria allowed for intention-to-treat analysis of the survival outcomes. Reporting by intention-to-treat reflects actual clinical practice and outcomes, because it allows for non-compliance and protocol deviations, increasing the generalizability of the results (43). This reduces potential bias of the treatment effect, because the study population is not limited to patients that received planned treatment such as surgery or adjuvant chemotherapy. Without the intention-to-treat analysis, a selection of patients with better outcomes due to immortal time bias is likely to occur (44). This meta-analysis of 38 studies comprising 3843 (B)RPC patients found superior survival following any neoadjuvant treatment compared to upfront resection (weighted median OS: 19 vs. 15 months). Only a negligible number of patients received neoadjuvant FOLFIRINOX. The resection rate was higher with upfront surgery (66 vs. 81%, *p* < 0.001), but the R0 resection rate was better after neoadjuvant treatment (87 vs. 67%, *p* < 0.001).

**Table 2:** Meta-analyses on neoadjuvant treatment for (borderline) resectable pancreatic cancer.

**References**	**No. studies**	**No. patients**	**No. (B)RPC**	**Treatment**	**Stage(s)**	**OS in months (95% CI)**	**Resection % (95% CI)**	**R0 resection % (of resected) (95% CI)**
Gillen et al. (2010) (42)	111	4,394	NR	Any preoperative treatment^*^	Resectable	23 (12–54)′ 8 (6–14)″	74 (66–81)	82 (73–90)
					BRPC/LAPC	21 (9–62)′ 10 (6–21)″	33 (26–41)	79 (72–85)
Dhir et al. (2017) (37)	96	5,520	2193	Any preoperative treatment^**^	Resectable	18 (13–28)	76 (68–84)	88 (80–94)
					BRPC	19 (9–45)	69 (59–78)	84 (67–96)
Versteijne et al. (2018) (25)	38	3,484	1738	Any neoadjuvant treatment^***^	Resectable	18 (10–50)	67 (64–70)	85 (NR)
					BRPC	19 (11–32)	65 (62–68)	89 (NR)
Janssen et al. (2019) (39)	20	283	283	FOLFIRINOX ± (chemo)radiotherapy	BRPC	22 (19–26)^‴^	68 (60–75)	84 (77–89)

*No., number; (B)RPC, (borderline) resectable pancreatic cancer; BRPC, borderline resectable pancreatic cancer; CI, confidence interval; LAPC, locally advanced pancreatic cancer; OS, Overall Survival*.

* *Neoadjuvant chemotherapy in 96% of studies, main agents gemcitabine, 5-FU, mitomycin C, and platinum compounds. Neoadjuvant radiotherapy in 94% of studies with doses ranging 24–63 Gy*.

** *Main chemotherapy agents FOLFIRINOX (810 patients), gemcitabine/taxane/capecitabine (410 patients), other three-drug regimens (60 patients), two-drug regimens (1,112 patients), single drug gemcitabine/5-FU/capecitabine (1,521 patients)*.

*** *All studies used at least chemotherapy as neoadjuvant treatment, including gemcitabine in 26 of 35 studies. Radiotherapy was given in 29 of 35 studies. No study used radiotherapy as sole neoadjuvant treatment. ′Resected. ″Not-resected.‴ Based on patient-level data*.

Following the ACCORD-11/PRODIGE-4 trial for metastatic PDAC by Conroy et al. in 2011, FOLFIRINOX emerged as a potential preoperative treatment for non-metastatic PDAC (3). No RCT has been performed for neoadjuvant FOLFIRINOX in the setting of (B)RPC. The best available estimate for the outcomes of patients treated with neoadjuvant FOLFIRINOX comes from a patient-level meta-analysis by Janssen et al. that included 283 BRPC patients and showed a median OS of 22.2 months (39). The pooled resection rate was 68%, with an R0 resection rate of 84%.

### Neoadjuvant Treatment—Large Retrospective Series

In addition to these meta-analyses, two large retrospective studies investigated the neoadjuvant approach (45, 46). The largest retrospective study used data from the National Cancer Database (NCDB) including patients with clinical stage I and II resected PDAC (45). A propensity score matched analysis was conducted comparing outcomes for patients who received neoadjuvant treatment before resection (*n* = 2005) to patients who underwent upfront resection (*n* = 6015). The neoadjuvant patients had a longer median OS compared to patients who underwent upfront resection (26 vs. 21 months, adjusted HR 0.72, 95% CI: 0.68–0.78, *p* < 0.01). Moreover, compared with a subgroup of patients who received adjuvant therapy after upfront resection, the neoadjuvant group still had better survival (26 vs. 23 months, adjusted HR 0.83, 95% CI: 0.73–0.89, *p* < 0.01). Second, a large observational cohort study from Verona Hospital included all consecutive BRPC (*n* = 267) and LAPC (*n* = 413) patients (46). Of all patients with newly diagnosed BRPC or LAPC, 7% received only supportive care owing to clinical deterioration. FOLFIRINOX (46%) and gemcitabine with *nab*-paclitaxel (22%) were the most commonly used regimens, and additional radiotherapy was applied in 23% of patients. Resection rate was 24% for BRPC patients, with an R0 resection rate of 58% for all patients combined. No differences were found in R0 resection rates between BRPC and LAPC patients and chemotherapy regimens used.

### Published Neoadjuvant FOLFIRINOX Trials (Phase II and III)

Three non-randomized small (<50 patients) phase II studies on neoadjuvant FOLFIRINOX for (B)RPC have been published to date (Table 3A) (47–49). In 2016, the first prospective multicenter trial was published (ALLIANCE A021101), including 22 BRPC patients who received preoperative mFOLFIRINOX (4 cycles) followed by capecitabine-based chemoradiotherapy (50.4 Gy in 28 fractions) (47). This study demonstrated the feasibility of recruiting patients in a multi-institutional neoadjuvant FOLFIRINOX study. Fifteen patients (68%) completed the neoadjuvant treatment and underwent a resection, with an R0 resection rate of 93%. The median OS was 22 months. In 2018, a similar study was published to determine the tolerability and efficacy of four cycles of mFOLFIRINOX both pre- and post-operative in *resectable* PDAC (48). Twenty-one patients were included, of whom 81% underwent a resection with an R0 resection rate of 94%. Following resection, 82% of patients completed 4 cycles of adjuvant mFOLFIRINOX. The largest study was a single-arm phase II clinical trial conducted at the Massachusetts General Hospital (49). In this study, 48 BRPC patients were treated with 8 cycles of neoadjuvant FOLFIRINOX followed by individualized chemoradiotherapy. In patients with resolution of vascular involvement, FOLFIRINOX was followed by short-course capecitabine-based chemoradiotherapy (25 Gy in 5 fractions), whilst patients with persistent vascular involvement were treated with long-course chemoradiotherapy (50.4 0Gy in 28 fractions). Forty-four patients (92%) proceeded to chemoradiotherapy, of whom 27 (56%) received short-course chemoradiotherapy and 17 (35%) received long-course chemoradiotherapy. Surgical resection was performed in 32 (67%) patients, of whom 31 (97%) had an R0 resection. After a median follow-up of 18 months, median OS was 38 months, with a 2-year OS of 56% (NCT0591733).

**Table 3:** Recently published neoadjuvant trials in (borderline) resectable pancreatic cancer from 2016 to 2019.

**Trial (year)**	**Sample size**	**Stage**	**Criteria**	**Treatment regimen (cycles)**	**Comparator (cycles)**	**Survival (*p*–value)**	**Resection (*p*–value)**	**R0 resection (*p*–value)**
**A. NEOADJUVANT FOLFIRINOX**								
**Non-randomized studies**								
ALLIANCE A021101 (2016) (47)	22	BRPC	Intergroup	Neoadj. mFOLFIRINOX(4) + capecitabine-based CRT	–	Median OS: 22 mo	68%	93%′
De Marsh et al. (2018) (48)	21	Resectable	NCCN	Periop. mFOLFIRINOX(4+4)	–	Median OS: 36 mo (resected only)	81%	94%″
Murphy et al. (2018) (49)	48	BRPC	NR	Neoadj. FOLFIRINOX(8) + short-course or long-course capecitabine-based CRT	–	Median OS: 38 mo	67%	97%′
**B. NEOADJUVANT REGIMENS OTHER THAN FOLFIRINOX**								
**Randomized trials**								
Golcher et al. (2015) (50)	73^*^	Resectable	<180° arterial or venous contact	Neoadj. gemcitabine+cisplatin based CRT + adj. gemcitabine(6)	Surgery + adj. gemcitabine(6)	Median OS: 17 vs. 14 mo (*p* = 0.96)^*^	58 vs. 70% (*p* = 0.31)^*^	89 vs. 70% (*p* = 0.81)^*^^nr^
PACT-15 (2018) (51)	93	Resectable	No vascular contact	c. Periop. PEXG(3+3)	a. Surgery + adj. gemcitabine(6) b. Surgery + adj. PEXG(6)	Median OS: 38 (c) vs. 20 (a) vs. 26 (b) mo (NR) 1-YR DFS: 66% (c) vs. 23% (a) vs. 50% (b) (NR)	84 (c) vs. 85 (a) vs. 90% (b) (NR)	63 (c) vs. 27 (a) vs. 37% (b) (NR)^nr^
Jang et al. (2018) (52)	50	BRPC	NCCN	Neoadj. gemcitabine-based CRT + adj. gemcitabine(4)	Surgery + adj. gemcitabine-based CRT+ gemcitabine(4)	Median OS: 21 vs. 12 mo (*p =* 0.028) 2-YR OS: 41 vs. 26%	63 vs. 78% (NR)	52 vs. 26% (*p* = 0.004)^‴^
PREOPANC-1 (2018) (53)	246	(B)RPC	DPCG	Neoadj. gemcitabine-based CRT(3) + adj. gemcitabine(4)	Surgery + adj. gemcitabine(6)	Median OS: 17 vs. 14 mo^**^ (*p* = 0.07) Median DFS: 10 vs. 8 mo^**^ (*p* = 0.02)	60 vs. 72%^**^ (*p* = 0.065)	63 vs. 31%^**^ (*p* < 0.001)^‴^
Preop-02/JSAP-05 (2019) (54)	364	Resectable	NR	Neoadj. S-1 + gemcitabine(2) + adj. S-1(6 mo)	Surgery + adj. S-1(6 mo)	Median OS: 37 vs. 27 mo (*p* = 0.015)	NR^***^	NR^***^^nr^
**Non-randomized studies**								
Tsai et al. (2018) (55)	130	(B)RPC	<180° SMA or CA, short segment abutment HA, venous reconstructable	Neoadj. 5-FU- or gemcitabine-based chemo(radio)therapy (8 w), depending on molecular profiling	–	Median OS: 38 mo 80% 5-FU-based 20% gemcitabine-based	82%	81%″
ACOSOG Z5041 (2018) (56)	114	Resectable	No arterial contact, <180° venous contact, no occlusion	Periop. gemcitabine + erlotinib (2+2)	–	Median OS: 21 mo 2-YR OS: 40%	73%	81%^nr^
JASPAC-05 (2019) (57)	52	BRPC	<180° SMA, CHA, or CA. Bilateral impingement of SMV/PV.	Neoadj. S1-based CRT	–	Median OS: 26 mo 2-YR OS: 51%	NR^**^	52%^nr^

*FOLFIRINOX, folinic acid + irinotecan + oxaliplatin + leucovorin; BRPC, borderline resectable pancreatic cancer; LAPC, locally advanced pancreatic cancer; NCCN, National Comprehensive Cancer Network; DPCG, Dutch Pancreatic Cancer Group; NR, not reported; DFS, disease free survival; OS, overall survival; Neoadj., neoadjuvant; Adj., adjuvant; Periop., perioperative; CRT, chemoradiotherapy; mo, months; d, days; PEXG, cisplatin, epirubicin, gemcitabine, and capecitabine; YR, year; YRS, year survival; SMA, superior mesenteric artery; CHA, common hepatic artery; CA, celiac artery; SMV, superior mesenteric vein; PV, portal vein*.

* *Results after early termination of the trial due to slow accrual*.

** *Results at interim analysis, after 85% of events needed*.

*** *Not reported in abstract, paper not yet published. ′R1 if microscopic tumor at any margin. ″R1 if microscopic tumor at SMA margin, common bile/hepatic duct or pancreatic transaction margins. ^‴^R1 if microscopic tumor <1 mm of any surface or margin. ^nr^definition of resection margin not reported*.

Although, the three studies slightly differ in the treatment regimen and sequence, neoadjuvant (m)FOLFIRINOX treatment with or without chemoradiotherapy is feasible with high R0 resection rates. The survival estimates are promising, but need confirmation in larger RCT's.

### Published Neoadjuvant Trials—Regimens Other Than FOLFIRINOX (Phase II and III)

A number of phase II–III trials have been conducted using other neoadjuvant regimens, yet several of these RCTs were terminated early due to slow accrual. This emphasizes the difficulties in conducting large neoadjuvant RCTs in pancreatic cancer. Table 3B shows eight published studies on neoadjuvant regimens other than FOLFIRINOX. Three RCTs have been published on neoadjuvant gemcitabine-based chemoradiotherapy vs. upfront surgery for patients with (B)RPC (50, 52, 53). The study by Golcher et al. was terminated early due to slow accrual after inclusion of 73 (29%) patients (50). They concluded that neoadjuvant chemoradiation is safe with respect to toxicity, postioperative morbidity, and mortality, but no difference in OS could be demonstrated (median OS: 17 vs. 14 months, *p* = 0.96). In the Korean randomized phase II-III trial, BRPC patients were randomly assigned to receive gemcitabine-based chemoradiotherapy (45 Gy in 25 fractions and 9 Gy in 5 fractions) (arm A) or upfront surgery followed by chemoradiotherapy following the same protocol as the neoadjuvant group (arm B) (52). Both groups received 4 cycles of gemcitabine as maintenance chemotherapy after completion of initial treatment. After inclusion of 50 patients, interim-analysis showed superior median OS (21 vs. 12 months, HR = 1.97, 95% CI: 1.07–3.62, *p* = 0.028), better 2-year survival rate (41 vs. 26%), and a superior R0 resection rate (52 vs. 26%, *p* = 0.004) in the neoadjuvant group compared to upfront surgery. Consequently, the study was discontinued due to superiority and lack of equipoise (NCT01458717). At ASCO 2018, the Dutch phase III PREOPANC-1 trial presented preliminary results, after inclusion of 246 (B)RPC patients who were randomly allocated to neoadjuvant gemcitabine-based chemoradiotherapy followed by a resection and adjuvant 4 cycles of gemcitabine (arm A), or upfront surgery followed by 6 cycles of gemcitabine (arm B) (53). After 85% of events needed, the interim analysis showed superior R0 resection rate (63 vs. 31%, *p* < 0.001) and superior DFS (10 vs. 8 months, *p* = 0.02) in the neoadjuvant group, but a difference in OS could not be demonstrated (17 vs. 14 months, HR = 0.74, *p* = 0.07). To allow for comparison with adjuvant trials, a subgroup analysis was performed of patients who received at least one cycle of adjuvant chemotherapy, showing a median OS of 42 months in the neoadjuvant group and 19 months in the upfront surgery group (*p* = 0.006). Final results are awaited soon. The PACT-15 trial was an Italian multicenter phase II trial, in which 93 *resectable* PDAC patients were randomly assigned (1:1:1) to receive adjuvant gemcitabine (arm A), adjuvant PEXG (cisplatin, epirubicin, gemcitabine, and capecitabine) (arm B), or 3 cycles of PEXG pre- and postoperative (arm C) (51). Median OS was 20 months in arm A, 26 months in arm B, and 38 months in arm C (*p*-value not reported). Three non-randomized studies on regimens other than FOLFIRINOX have been published (55–57). The phase II trial from Tsai et al. used molecular profiling of pretreatment EUS-FNA guided tumor biopsies using 6 biomarkers to guide neoadjuvant therapy in 130 (B)RPC patients (55). Eighty percent of patients received 5-FU based treatment whilst 20% received gemcitabine-based treatment. The median OS was 38 months, with a 5-year survival of 34%, a resection rate of 82%, and an R0 resection rate of 81%. The ACOSOG Z5401 single-arm phase II trial was a study of neoadjuvant gemcitabine plus erlotinib for *resectable* PDAC. (56) This study demonstrated a favorable 2-year OS for 114 evaluable patients of 40% (95% CI: 31–49%), with a median OS of 21 months. At the 2019 ASCO congress, final results of two Japanese trial were presented. The JASPAC-05 study was a multicenter, single-arm, phase II of neoadjuvant S-1 based chemoradiotherapy (57). Fifty-two BRPC patients were included, and 50 (96%) patients completed the neoadjuvant treatment. The 2-year OS was 51%, with a median OS of 26 months, and an R0 resection rate of 52%. The phase II-III Preop-02/JSAP-05 trial was a large collaboration study of 57 centers in which 364 patients with *resectable* PDAC were randomized to either neoadjuvant gemcitabine and S-1 chemotherapy (2 cycles) or upfront surgery, both followed by 6 months of adjuvant S-1 (54). This study also showed superior survival following neoadjuvant treatment, with a median OS of 37 vs. 27 months (HR = 0.72, 95% CI: 0.55–0.94, *p* = 0.015). No differences were found regarding the resection rate, R0 resection rate, and postoperative morbidity. Although S-1 is only used as standard-of-care in East Asia, the study does provide additional proof of the superiority of neoadjuvant therapy over upfront resection for patients with *resectable* PDAC.

In summary, although based on only three RCTs, a neoadjuvant approach seems to be consistently superior to upfront resection for R0 resection rates, at least equal or superior for DFS, and at least equal or superior for OS in both BRPC and *resectable* PDAC patients. The results of the R0 resection rates were notable, with a two-fold increase in two out of the three evaluable RCTs. However, it remains unclear whether superior R0 resection rate is an appropriate intermediate outcome for OS in the neoadjuvant setting. The results of ongoing larger RCTs may further clarify the survival benefit of neoadjuvant treatment as opposed to upfront resection for (B)RPC patients.

### Standard of Care—Current Guidelines

The NCCN guideline, ASCO Clinical Practice Guideline, and European Society for Medical Oncology (ESMO) Clinical Practice Guideline are commonly used guidelines for pancreatic cancer treatment (15, 21, 58, 59). Due to the lack of large RCTs for neoadjuvant treatment of PDAC, most recommendations in these guidelines are based on systematic reviews of cohort studies, providing Oxford Levels of Evidence category 2A (60).

The 2019 NCCN guidelines (15) recommend upfront surgery followed by adjuvant treatment for *resectable* PDAC, but advise to consider neoadjuvant treatment in patients with high-risk features, preferably in the setting of a clinical trial. High-risk features include imaging findings suspicious of advanced or metastatic disease, significantly elevated Carcinogen Antigen (CA) 19-9, large primary tumors or regional lymph nodes, excessive weight loss, and notable pain. The adjuvant treatment of first choice is mFOLFIRINOX. For BRPC patients, neoadjuvant treatment is recommended, with therapeutic options including FOLFIRINOX or gemcitabine/*nab*-paclitaxel, both with or without subsequent chemoradiotherapy. The 2019 ASCO Clinical Practice Guideline (21) recommends primary surgical resection for patients without any radiographic evidence of metastatic disease, with no interface between the primary tumor and surrounding mesenteric vasculature, CA 19.9 level suggestive of potentially curable disease, and a performance status and comorbidity profile appropriate for major abdominal surgery. However, neoadjuvant therapy can also be offered as an alternative strategy for patients with *resectable* PDAC. For patients who do not meet all of these criteria, the ASCO guideline recommends neoadjuvant therapy. No specific neoadjuvant treatment regimen is recommended. Options for consideration include FOLFIRINOX or gemcitabine/*nab*-paclitaxel ± subsequent chemoradiotherapy. In the adjuvant setting, mFOLFIRINOX is recommended as treatment of first choice. In case of concern for toxicity and tolerance, doublet therapy with gemcitabine and capecitabine, or monotherapy with either gemcitabine or fluorouracil (5-FU) can be offered. Following neoadjuvant therapy, patients may be candidates for additional chemotherapy following surgery, depending on their performance status and initial response to the neoadjuvant treatment. The ASCO guideline recommends a total of 6 months of chemotherapy, considering both neoadjuvant and adjuvant treatment. Adjuvant chemoradiotherapy may be offered to patients who underwent primary resection with microscopically positive margins (R1) and/or node-positive disease after completion of systemic adjuvant chemotherapy. The 2019 ESMO guideline (58, 59) recommends adjuvant mFOLFIRINOX as first therapeutic option in selected and fit individuals with resectable tumors. For patients with age >70 years, WHO performance status 2, or patients who have any contraindication for FOLFIRINOX, doublet therapy with gemcitabine-capecitabine can be offered as alternative. Gemcitabine monotherapy should be used only in frail patients. For BRPC patients, neoadjuvant treatment with gemcitabine or FOLFIRINOX followed by chemoradiotherapy and surgery is recommended.

### Ongoing Neoadjuvant FOLFIRINOX Trials (Phase II and III)

The optimal chemotherapy regimen in the neoadjuvant setting, the number of cycles pre- and postoperatively, the additional benefit of (chemo)radiotherapy, and the timing of surgery after neoadjuvant treatment still need to be further investigated. Several ongoing phase II and III trials are investigating these aspects of neoadjuvant treatment regimens in patients with (B)RPC. Table 4A presents selected ongoing trials including neoadjuvant FOLFIRINOX, and Table 4B shows ongoing trials for neoadjuvant regimens other than FOLFIRINOX.

**Table 4:** Ongoing neoadjuvant trials for (borderline) resectable pancreatic cancer.

**Trial**	**Sample size**	**Stage**	**Criteria**	**Treatment regimen (cycles)**	**Comparator (cycles)**	**Primary outcome**	**Start**	Status^**^
**A. NEOADJUVANT FOLFIRINOX**								
**Randomized trials**								
ESPAC-5F ISRCTN89500674	85	BRPC	NR	a. Neoadj. FOLFIRINOX(4) b. Neoadj. gemcitabine(1)+ capecitabine(2)	c. Neoadj. capecitabine-based CRT d. Surgery + adj. gemcitabine(6) or 5-FU(6)	Recruitment, R0 resection rate	04-2014	Results pending
NEPAFOX NCT02172976	40	(B)RPC	Venous reconstructable, no contact SMA or CA	Periop. FOLFIRINOX (4-6 + 4-6)	Surgery + adj. gemcitabine(6)	OS	11-2014	Results pending
SWOG S1505 NCT02562716	112	Resectable	<180° venous, no arterial	Periop. mFOLFIRINOX (3 + 3)	Periop. gemcitabine/*nab*-paclitaxel (3 + 3)	OS at 2-yr	10-2015	Results pending
NorPACT-1 NCT02919787	90	Resectable	NCCN	Neoadj. FOLFIRINOX(4) + adj. gemcitabine-capecitabine(4)	Surgery + adj. gemcitabine-capecitabine(6)	OS at 1-yr (resected only)	09-2016	Recruiting
PANDAS- PRODIGE 44 NCT02676349	90	BRPC	NCCN	Neoadj. mFOLFIRINOX + capecitabine-based CRT + adj. gemcitabine or mLV5FU	Neoadj. mFOLFIRINOX + adj. gemcitabine or mLV5FU	R0 resection rate	10-2016	Recruiting
ALLIANCE A021501 NCT02839343	134	BRPC	Intergroup	Neoadj. FOLFIRINOX(8) + adj. mFOLFOX6(4)	Neoadj. mFOLFIRINOX(7) + SBRT + adj. FOLFOX(4)	OS at 1.5-yr	12-2016	Suspended (interim analysis)
PANACHE01- PRODIGE48 NCT02959879	160	Resectable	NCCN	a. Neoadj. mFOLFIRINOX(4) + adj chemotherapy(8) b. Neoadj. FOLFOX(4) + adj. chemotherapy(8)	c. Surgery + adj. chemotherapy(12)	OS at 1-yr	03-2017	Recruiting
PREOPANC-2 NTR7292	368	(B)RPC	DPCG	Neoadj. FOLFIRINOX(8)	Neoadj. gemcitabine-based CRT(3) + adj. gemcitabine(4)	OS	06-2018	Recruiting
ALLIANCE A021806	344	Resectable	<180° venous, patent confluence, no arterial	Periop. mFOLFIRINOX (8 + 4)	Surgery + adj. mFOLFIRINOX (12)	OS	2020	Start recruiting 2020
**Non-randomized studies**								
Lacy et al., Yale NCT02047474	46	Resectable	No venous occlusion/encasement, no arterial	Periop. mFOLFIRINOX (6 + 6)	–	PFS at 1-yr	09-2013	Recruiting
IUCRO-0473 NCT02178709	48	Resectable	NR	Neoadj. FOLFIRINOX(4)	–	Pathological complete response^*^	04-2014	Recruiting
**B. NEOADJUVANT REGIMENS OTHER THAN FOLFIRINOX**								
**Randomized trials**								
UVA-PC-PD101 NCT02305186	56	(B)RPC	NR	Neoadj. pembrolizumab + capecitabine-based CRT	Neoadj. capecitabine-based CRT	Toxicity, TILs	03-2015	Recruiting
Laheru et al. Johns Hopkins NCT00727441	87	Resectable	No contact SMA/CA, patent SMV/PV	Periop. GVAX(1+5) + neoadj. cyclophosphamide iv (a) or oral (b) + adj. CRT	c. Periop. GVAX(1+5) + adj. CRT	Safety, feasibility, immune response	03-2015	Final results pending
NEONAX NCT02047513	166	Resectable	No arterial contact	Periop. gemcitabine/*nab*-paclitaxel(2+4)	Surgery + adj. gemcitabine/*nab*-paclitaxel(6)	DFS	04-2015	Recruiting
**Non-randomized studies**								
Park et al. National Cancer Center Korea NCT01333124	64	Resectable	NR	Neoadj. gemcitabine-based CRT	–	R0 resection rate	04-2014	Recruiting
Okada et al. Wakayama NCT02926183	60	BRPC	NCCN	Neoadj. gemcitabine/*nab*-paclitaxel(2)	–	OS	10-2016	Recruiting
PRO30720 NCT03322995	125	(B)RPC	NR	Adaptive modification of neoadj. treatment based on clinical response + CRT^***^	–	Completion neoadj. regimen incl. resection	06-2018	Recruiting
ESR-16-12315 NCT03572400	71	(B)RPC	Stage I or II AJCC 8th	Neoadj. gemcitabine/dervalumab-based CRT(6) + adj. gemcitabine/dervalumab(6) + dervalumab(12 mo)	–	DFS	11-2018	Recruiting

*(B)RPC, (borderline) resectable pancreatic cancer; BRPC, borderline resectable pancreatic cancer; NCCN, National Comprehensive Cancer Network; AJCC, American Joint Committee on Cancer; NR, not reported; DFS, disease-free survival; OS, overall survival; Neoadj., neoadjuvant; Adj., adjuvant; Periop., perioperative; CRT, chemoradiotherapy; IORT, intraoperative radiation therapy; SBRT, stereotactic body radiation therapy; FOLFIRINOX, folinic acid + irinotecan + oxaliplatin + leucovorin; mFOLFIRINOX, modified FOLFIRINOX; FOLFOX6, folinic acid + leucovorin + oxaliplatin; mLV5FU, modified folicin acid + leucovorin; PV, portal vein; SMA, superior mesenteric artery; CA, celiac artery; Incl., including; mo, months; Vs., versus; RCT, randomized controlled trial; TIL, tumor-infiltrating lymphocytes*.

* *Evaluated by MRI or CT*.

** *Based on clinicaltrials.gov, assessed on 21-08-2019*.

*** *Adaptive modification of neoadjuvant therapy: after 2 months, first-line chemotherapy is continued in responders, changed to second-line therapy in patients with stable disease, or changed to chemoradiation in patients with local disease progression. After 4 months of chemotherapy, patients will be treated with chemoradiotherapy. Patients who underwent a resection after receiving <4 months of neoadjuvant chemotherapy, will be offered adjuvant therapy at the discretion of their physician*.

Of the nine RCTs, two originate from France: the PANDAS-PRODIGE44 trial for BRPC patients, and the PANACHE01-PRODIGE48 trial for *resectable* PDAC. In the PANDAS-PRODIGE44 trial, 90 BRPC patients will receive neoadjuvant mFOLFIRINOX with (arm A) or without capecitabine-based chemoradiotherapy (arm B), both followed by surgery and adjuvant gemcitabine or modified LV5FU (NCT02676349). This study uses R0 resection rate as primary endpoint. The PANACHE01-PRODIGE48 is a three-arm trial with 2:2:1 allocation to 4 cycles of neoadjuvant mFOLFIRINOX (arm A) or FOLFOX (arm B), both followed by 8 cycles of adjuvant chemotherapy, or upfront surgery followed by 12 cycles of adjuvant chemotherapy (arm C) (NCT02959879) (61). The choice of adjuvant chemotherapy regimen will be left to the medical teams, according to guidelines during the recruitment period. The trial will include 160 *resectable* PDAC patients, and the primary endpoint is 1-year OS. The SWOG S1505 trial is a randomized phase II study for patients with *resectable* PDAC designed to determine the most promising perioperative regimen for a larger phase III trial (NCT02562716). This study has completed accrual and randomized 147 patients to either 3 cycles of perioperative mFOLFIRINOX (arm A) or perioperative gemcitabine with *nab*-paclitaxel (arm B). The primary outcome is 2-year OS, and results are anticipated in 2020. The ALLIANCE A021501 was initially designed to evaluate the additional value of hypofractionated radiation therapy to systemic therapy as neoadjuvant treatment for BRPC of the pancreatic head, with 18-month OS rate as primary outcome(NCT02839343). (62) The initial design of this study was to randomize 134 patients to receive 8 cycles of mFOLFIRINOX (arm A), or 7 cycles of mFOLFIRINOX followed by either hypofractionated stereotactic body radiation therapy (SBRT, 33 Gy in 5 fractions) or hypofractionated image guided radiation therapy (HIGRT, 25 Gy in 5 fractions) (arm B). Following surgery, all patients were scheduled for 4 cycles of adjuvant modified FOLFOX6 (mFOLFOX6). However, an interim analysis of the R0 resection rate was conducted after accrual of 30 patients, after which the radiotherapy arm (B) was suspended due to futility. The NorPACT-1 trial is a multicenter trial for patients with *resectable* PDAC of the pancreatic head, in which patients are randomized in a 3:2 ratio to receive 4 cycles of neoadjuvant FOLFIRINOX and adjuvant 4 cycles of gemcitabine-capecitabine (arm A), or upfront surgery followed by 6 cycles of adjuvant gemcitabine-capecitabine (arm B) (NCT02919787) (63). The sample size is 90 patients, and the primary endpoint is 1-year OS for those patients who ultimately undergo a resection. The PREOPANC-2 trial is a multicenter study performed by the Dutch Pancreatic Cancer Group (DPCG) (NTR7292) (64). In this study, 368 (B)RPC patients will be randomized to receive 8 cycles of neoadjuvant FOLFIRINOX (arm A) or 3 cycles of neoadjuvant gemcitabine-based chemoradiotherapy with adjuvant 4 cycles of gemcitabine, with median OS as primary endpoint. Last, the ALLIANCE A021806 trial will compare 8 cycles of neoadjuvant and 4 cycles of adjuvant mFOLFIRINOX to all 12 cycles adjuvant mFOLFIRINOX for *resectable* PDAC. This trial will start recruiting patients by the beginning of 2020 and will include 344 patients using median OS as primary endpoint. The remaining three studies investigate neoadjuvant FOLFIRINOX with a sample size of <50 patients, thereby limiting potential impact on future guidelines [NCT02047474, NCT02178709, NCT02172976 (NEPAFOX)].

### Ongoing Neoadjuvant Trials—Regimens Other Than FOLFIRINOX (Phase II and III)

At least three ongoing randomized phase II-III trials (NCT02305186, NCT00727441, NCT02047513) and four ongoing single-arm phase II trials are investigating neoadjuvant regimens other than FOLFIRINOX (NCT01333124, NCT02926183, NCT03322995, NCT03572400) (Table 4B). The three-arm trial from Johns Hopkins aims to study the feasibility and toxicity of perioperative GVAX vaccine therapy ± cyclophosphamide (oral or intravenous) in addition to standard adjuvant chemoradiotherapy for *resectable* PDAC (NCT00727441). This study is awaiting final results. In the randomized NEONAX trial, 166 patients with *resectable* PDAC are randomized to receive 6 cycles of gemcitabine with *nab*-paclitaxel perioperative (2 neoadjuvant, 4 adjuvant) (arm A), or all cycles adjuvant (arm B) (65). In the PRO30720 study, the neoadjuvant regimen depends on the response on CT or MRI scan, tumor marker levels, and performance status assessment (NCT03322995). Sample size is 125 (B)RPC patients, who will all start with 2 months of neoadjuvant chemotherapy. Subsequent treatment depends on the response and may include a therapy switch to an alternative chemotherapy regimen or chemoradiotherapy. With this adaptive design, the feasibility of personalized treatment will be evaluated. The other ongoing trials comprise a variety of interventions including chemoradiotherapy (NCT02305186, NCT01333124, doublet chemotherapy (NCT02926183), and a combination of chemotherapy and immunotherapy (NCT03572400).

Most ongoing studies of both neoadjuvant FOLFIRINOX and other neoadjuvant regimens are underpowered to detect a clinically relevant difference (e.g., 3 or 6 months) in OS. Some studies are hypothesis-generating in their selection of intermediate outcome, such as R0 resection or treatment completion rates. Other studies do have survival as primary outcome, but have a sample size that is too small to detect a clinically relevant survival difference of 3 or 6 months. Assuming an alpha error of 0.05 and a power of 80%, a sample size exceeding 300 patients is needed to detect a difference in median OS of 6 months. An explanation for inadequate sample size is often a concern for feasibility. The PREOPANC-2 trial appears to be the only RCT that may be adequately powered to assess whether neoadjuvant FOLFIRINOX is superior to other regimens. Furthermore, the ALLIANCE A021806 is the only adequately powered RCT comparing perioperative (8 + 4 cycles) mFOLFIRINOX with adjuvant mFOLFIRINOX (12 cycles).

## Conclusion

Selection bias hampers comparing survival outcomes between neoadjuvant and adjuvant trials. Patients in neoadjuvant trials may have occult metastatic disease at surgery or may not fully recover from surgery; patients in adjuvant trials were selected after overcoming these hurdles. Only a direct comparison in an RCT will avoid this inevitable selection bias. Despite the limited number of published RCTs comparing a neoadjuvant approach to upfront surgery, patients with resectabel PDAC and BRPC seem to consistently benefit from a neoadjuvant approach with regards to the R0 resection rate. Furthermore, the DFS and OS were at least equal or superior with a neoadjuvant approach compared to upfront surgery. The currently published RCTs supporting neoadjuvant treatment over upfront resection included mostly single-agent based regimens. The multi-agent regimen FOLFIRINOX has considerable toxicity requiring a good performance status. FOLFIRINOX has already proven superior to gemcitabine in the metastatic and adjuvant setting. Ongoing RCTs will investigate whether FOLFIRINOX is indeed the superior regimen in the neoadjuvant setting. Likely, neoadjuvant FOLFIRINOX may further improve the outcomes of this vulnerable patient group. In addition, future RCTs should study the optimal number of neoadjuvant cycles, the value of additional neoadjuvant chemoradiotherapy, the optimal patient selection for surgical resection, and the need for subsequent adjuvant chemotherapy. For patients with a good performance status, we advocate patient participation in one of the large ongoing RCTs evaluating the potential benefit of neoadjuvant FOLFIRINOX for (B)RPC patients.

## Author Contributions

All authors listed have made a substantial, direct and intellectual contribution to the work, and approved it for publication.

### Conflict of Interest

EO'R received funding for the Memorial Sloan-Kettering Cancer Center for pancreatic cancer research in general from the following companies: Genentech, Roche, BMS, Halozyme, Celgene, ActaBiologica, OncoMed, Parker Institute, AstraZenica, Silenseed. Consulting/Advisory: Cytomx, BioLineRx, Targovax, Halozyme, Celgene, Bayer, Polaris. EO'R receives personal fees/consulting/advisory funds from Cytomx, BioLineRx, Targovax, Halozyme, Celgene, Bayer, Polaris. The remaining authors declare that the research was conducted in the absence of any commercial or financial relationships that could be construed as a potential conflict of interest. The handling editor declared a past co-authorship with one of the authors BK.
